# Cases of Preternatural Labour

**Published:** 1839-01-05

**Authors:** William Harris


### Cases of Preternatural Labour. *Reported by* William Harris, M. D.

### *Case First.*

A few weeks since, I was called to consult with a medical friend, in a case of preternatural labour. The patient was about thirty years of age, in robust health, and the mother of several children. She had been about twelve hours in the first stage of her labour, and it was now about fourteen hours since the second stage had commenced. She was not, however, exhausted; her spirits were good, and her pains powerfully expulsive. Upon examination, I found a hand outside of the vulva, two feet just within the orifice of the vagina, one a little in advance of the other, and a head engaged in the superior strait.

My first effort was to ascertain whether the head and feet belonged to the same child, or to different children. The head presented in the second position, the hand was a left hand, and the feet were in the fifth position. The relative positions of the different presenting parts led me to think that they all might belong to the same child, but I determined to investigate the case further. I attempted to disengage the head, and push it up into the right iliac fossa, but the membranes having been ruptured several hours, the liquor amnii all drained off, and the expulsive efforts powerful, the attempt was unsuccessful. I next endeavoured to satisfy myself by passing my hand up the legs, as far as the breach; but this effort was also impracticable.

What, under existing circumstances, was to be done? If there were two children, and traction efforts were made upon the legs now in the vagina, the head of the first child would be likely to lock upon the head of the second, now engaged in the superior strait. It was decided that the safer course was to bring down the head, and a difficulty here arose as to whether the child was dead or alive. If dead, it would be much easier to act upon the head, in its present situation, with the crotchet; but if alive, the forceps must be tried. The hand without the vulva was cold and pulseless, and the beating of the fœtal heart could not be heard; but, as the signs of fœtal death cannot be fully relied upon, I decided to try the forceps, and, accordingly, applied them without much difficulty. The head was now brought down, the feet descending before it, and the shins in contact with the face. The delivery was accomplished after a few minutes, and there proved to be but one child, which was dead; and, judging from the discolouration, had been so for several hours. The patient recovered from her accouchement very rapidly.

### *Case Second.*

In the course of last year I attended a lady in her accouchement, who had had several still-born children in succession, in consequence of malpresentations. She and her friends were, of course, exceedingly anxious that, on this occasion, she should be favoured with a living child. Under these circumstances, the reader may judge what were my feelings, when, upon examination, I discovered that the membranes were ruptured, and a large gelatinous umbilical cord presented, the fold of which was just within the vulva, and

---

had been evidently carried down by the gush of waters. Just above the superior strait, not yet engaged, was the breech, in the first position, with the heels in contact with the nates. The pulsation in the cord was strong, and the breech in a favourable position, as I hoped, for a successful manœuvre. I accordingly placed the funis between the second and index fingers of the left hand, carried it up in front of the child, above its knees, and lodged it safely between them and its abdomen. I, then, in withdrawing the hand, carefully seized the feet, the toes in the palm of the hand, and forefinger between the ankles, and, by a sudden traction effort, brought down the legs and thighs—the breech, now, plugging up the superior strait, and leaving the funis above. My anxiety induced me to proceed with a rapid and forcible delivery. My efforts were crowned with complete success, and I had the high gratification of presenting to a delighted mother a living child. Not more than ten minutes elapsed between the commencement of this manœuvre and the birth of the child. The mother recovered without an untoward symptom.

*Case Third.*

I was called, a few years since, to consult with a distinguished physician, in a very rare and interesting case of preternatural labour. The subject of these remarks was upwards of forty years of age, in good health, and pregnant with her first child.

The first stage of her labour was about ten hours. The second stage had continued twelve hours when I arrived. The patient was strong, by no means desponding, and her expulsive pains violent. Upon examination, I found the two hands of the fœtus outside the vulva, the thorax firmly engaged in the superior strait, the head turned backwards, with the vertex in contact with the back, and the chin resting upon the top of the symphysis pubis. I decided, at first, to attempt version by the feet; but the waters had been so long lost, and the expulsive efforts so violent, that this was utterly impracticable. As the fœtal heart had ceased to pulsate, and as the presenting hands were cold, pulseless, and discoloured, I recommended that the child be mutilated, and proposed its decapitation, to which the attending physician and mother readily assented. I had, at the time, in my pocket, a pruning knife, with a long handle, and a short, strong blade. I passed the blade carefully behind the neck, placed my thumb in front of it, and thus, safely to the mother, decapitated the child. I then took hold of the hands, and, without difficulty, delivered the body, and, afterwards, by introducing my forefinger into the mouth, the head was promptly brought down. The child appeared to have been dead several days; the practice was, therefore, clearly judicious,—and, if it had been alive, I doubt very much whether any other course, at that late period, would have succeeded. The mother recovered, enjoyed afterwards good health, and never had another child.

